# First Report on Successful Triploidy Induction in *Clarias gariepinus* (Burchell, 1822) Using Electroporation

**DOI:** 10.1038/s41598-020-59389-2

**Published:** 2020-02-12

**Authors:** Victor Tosin Okomoda, Lubna Aminath, Sunday Abraham Oladimeji, Ambok Bolong Abol-Munafi, Alabi Isaiah Korede, Mhd Ikhwanuddin, Joshua A. Umaru, Anuar Hassan, Chukwuemeka Onwuka Martins, Sheriff Md. Shahreza

**Affiliations:** 1 Department of Fisheries and Aquaculture, College of Forestry and Fisheries, University of Agriculture, P.M.B., 2373 Makurdi, Nigeria; 2 Institute of Tropical Aquaculture and Fisheries Research (AQUATROP), Universiti Malaysia Terengganu, 21030 Kuala Nerus Terengganu, Malaysia; 3 Faculty of Food Science and Fisheries, Universiti Malaysia Terengganu, 21030 Kuala Nerus Terengganu, Malaysia; 4 Agricultural Department, National Biotechnology Development Agency (NABDA), Abuja, Nigeria; 5 Department of Agricultural Extension and Management, Federal College of Forestry, Jos. Plateau, Nigeria; 6 Fisheries Technology Department, College of Agriculture Lafia, Nassarawa State, Nigeria; 7 Faculty of Forestry, Universiti Putra Malaysia, 43400 Serdang, Selangor Malaysia

**Keywords:** Animal breeding, Animal biotechnology

## Abstract

This study investigated the use of electric-shock in inducing triploidy in African catfish *Clarias gariepinus*. To achieve this, three voltages (9, 12, 21 V) were applied for different durations (3, 5, 10 min). The shock was initiated approximately three minutes after fertilization followed by incubation in ambient temperature. After incubation, hatchability and survival rates were determined while ploidy status of the treatment fishes was confirmed in one-month-old fingerlings using the exclusive triploid range of the erythrocyte major axis previously reported for the same species (11.9–14.9 μm) and by cytogenetic analysis of the chromosome. The results showed triploidy were achieved in 10 to 85% of the treatment groups. A consistent trend of decrease in hatchability and an increase in triploidy rate was observed with increased electroporation voltages and shock durations. The mean erythrocyte major axis length of triploid progenies (3n = 84) was observed to be between 11.3–14.6 μm and was higher than the range of 7.0–10.5 μm recorded for diploid progenies (2n = 56). It was concluded that electric shock can be used to induce triploidy in African catfish *C. gariepinus*.

## Introduction

Aquaculture growth is predicated on the need to feed an ever-growing population, hence the development and application of modern biotechnological tools to improve production characteristics of fishes. Today, aquaculture is prided as the fastest-growing animal food-producing sector in the world^1^. This progress has been made possible through the accumulation of knowledge on the biology of several fish species and its uses to develop advanced modern technology^2^. For instance, the degeneration of three of four meiotic products during the female gamete development in animals causes the extrusion of two polar bodies^3^. The understanding of this process has made the artificial induction of polyploidy possible by simply preventing the escape of the polar bodies, hence suppressing the first or second meiotic division^4,5^.

In nature, many plants and animals have been found to have polyploid status, thereby leading to the phenomenon of gigantism^6^. Polyploidy in the wild has been said to be as a result of the occasional failure of the extrusion of the second polar body in wild fertilized eggs^7^ consequent upon environmental changes or hybrid stabilization^8^. Hence, polyploidy occurs in a wide variety of organisms including plants, insects, mollusk, crustaceans, amphibians, reptiles, fish and mammals^9^. This has increased the curiosity of scientists to the possibility of artificially inducing polyploidy in cultured agricultural animals. Artificial chromosome manipulation techniques were at first developed with amphibians^10^ but later proved to be well suited for other aquatic organisms^11^. Today, there are archives of research on the application of chromosome manipulation techniques in many finfish species using diverse shock treatments^12–14^.

However, among all chromosome manipulation techniques, the induction of triploidy is one of the best ways of producing sterile fishes^4,5,15^. Although the application of a high dosage of steroid hormones have been reported to produce the same effect^16^; however, triploid fish are preferred for consumption over hormone-treated fish^17^. The advantages of sterility in triploid fish are more obvious when the cultivation period of the fish extends beyond sexual maturation. This is because sterility causes energy needed for gamete production to be channel into somatic growth^18,19^. Consequently, this will improve the fish’s flesh quality, reduce mortality and prevent fish reproduction thereby minimizing the possible impact of genetic and ecological disorder linked to the interactions between wild and cultured fishes^11^.

There are different techniques for the induction of triploids; some of which have been well described in many previous studies^4,5,15,20^. The various means for suppressing the second meiotic division may include temperature shock (heat and cold), pressure shock, chemical shock, and some anesthetics as well as electric shock^21^. Although many of these methods have proved effective in different fishes, however, each method is not without its pros and cons. Temperature seems to be the most commonly applied shocks for chromosome manipulation largely because of its simplicity, inexpensive nature and its scalability capability for mass production^22^. However, it is less reliable to give consistent and precise results, probably due to the difficulty in applying a controlled temperature homogenously on an egg batch^4,5^. Pressure shock, on the other hand, has been known to give much precise results, 100% triploidy induction success and reduce larvae mortality; however, it requires more expensive equipment for induction to be achieved^15^. Chemicals and anesthetics have also been reported to suppress the second mitotic division in several shellfishes but they are not commonly used in fin fishes^5^. Electroporation appears to be the least common means of polyploid induction^21^. Only a few studies have reported findings in oyster *Crassostrea gigas*, mussels (*Mytilus edulis* and *M. galloprovincialis*)^23^, coho salmon *Oncorhynchus kisutch*^24,25^ and red hybrid tilapia (*Oreochromis mossambicus* × *Oreochromis niloticus*)^19^. Hassan *et al*.^19^, had earlier stated that electroporation could be a much viable commercial alternative to other shock protocol if optimization is achieved. However, the optimization of shock processes is quite complicated by a large number of variables involved. These include the time of shock initiation after fertilization, the intensity of shock and duration of the shock to be applied^4,12,26^.

Among the different aquaculture candidates of the world, the African catfish *Clarias gariepinus* is considered one of the most excellent animal models for genetic and developmental studies^27^. It is popularly cultured in West Africa, South East Asia and many parts of the world^28^. Successful triploidization has been reported in many previous studies using temperature shock^13,14,29^. The advantages of triploidy induction in the African catfish *C. gariepinus* include but not limited to higher post-maturity weight gained, better body composition, higher gutted weight, and revenue from sales^30,31^. The time window in which triploidy can be induced (i.e. the extrusion of the second polar body) as reported in many previous studies has been given to be between 3 to 4 min post-fertilization. However, to our knowledge, no study on the African catfish has attempted to induce triploidization using electroporation. This study is therefore aimed at determining the possibility of triploidy induction in African catfish *C. gariepinus* using different electric voltages and shock duration.

## Materials and Methods

Twelve sexually mature broodstocks of *C. gariepinus* (sex ratio 1:1) weighing between 1 kg–1.5 kg were obtained from well-known fish farms around the environs of Terengganu in Malaysia. They were transported to and acclimatized at the Faculty of Food Science and Fisheries hatchery of the Universiti Malaysia Terengganu. The experimental protocols for this study were approved by the Universiti Malaysia Terengganu committee on research. All methods used in this study involving the care and use of animals were following international, national, and institutional guidelines.

Induced breeding of the catfish was performance following the methods of Hassan *et al*.^19^. The reported results in this study are combinations of data from three different breeding trials using two pairs of male and female brood fish at each instance. In brief, females were injected (using OVAPRIM at 0.5 ml kg^−1^) and allowed to swim for a latency period of nine hours in separate rearing tanks measuring 1 × 2 × 1 m^3^ each. The eggs were stripped from the females and collected in a clean bowl by applying gentle pressure along the abdomen of the fish. The male broodstocks were euthanized so the testis can be removed through laceration of the abdominal cavity using scissors. Fertilization was then done by mixing the eggs and milt from the testis, followed by activation with water.

The fertilized eggs were then quickly divided and 2 grams (i.e. 1000–1300 eggs) each were spread evenly (single layer of eggs) into the plastic strainers contained in the eleven bowls representing nine shock treatments and two controls (positive and negative control) designed for this study. The nine treatments used in the study were applications of three voltages (9, 12, 21 V) for three different shock durations (3, 5, 10 min). The electric field was supplied from ENERGIZER batteries (i.e. 1.5 V and 9 V volts). A 9 V battery was used to supply needed electric field to the specified treatments (i.e. eggs shocked with 9 V). To obtain 12 V in this study, a 9 V battery was connected in series with two number 1.5 V batteries. Similarly, the 21 V in this study was obtained from a series connection of two number 9 v batteries and two number 1.5 V batteries. The rectangular electric probes (positive and negative ends) each measuring 50 cm were placed in the opposite edges of the length of the eleven bowls (80 × 60 × 40 cm^3^ each) at about 3 cm below the highest water level (water depth = 30 cm) for the electroporation process. Each setup was monitored by a voltmeter and stopwatch to ensure the target voltage and specified time are maintained. Electroporation was initiated approximately three minutes post-fertilization which was perceived to be the time of extrusion of the second polar body as optimized in many previous studies^13,14,29^.

To facilitate the transmission of the electric field generated from the batteries, the water in the bowls was maintained at 5 ppt salinity level. This was done by diluting seawater with freshwater until 5 ppt is attained. The concept of positive control (+ve control) in this study was to determine if the 5 ppt salinity exposure in the different treatment groups had any effect on the outcome of this study^19^. Hence, eggs for the +ve control group were only maintained in a similar saline medium (i.e. 5 ppt) for the maximum duration of the shock process in the study (20 min), but not subjected to electroporation process. The negative control (−ve control) on the other hand was maintained in freshwater throughout the post-fertilization processes and development. Water temperature (27.55 ± 0.21 °C), pH 7.0 ± 0.31; Dissolved oxygen (5.75 ± 0.12 mg l^−1^) was optimum for the short durations of electric shock. After electroporation, the treated and control eggs were transferred into the incubation chambers (i.e. aquariums measuring 80 × 60 × 40 cm^3^ connected to a re-circulatory system) with freshwater for further development. The water quality of the incubation chambers in the re-circulatory system were maintained at optimum too (Temperature = 27 ± 0.14 °C; pH = 7.5 ± 0.12; Conductivity = 565 ± 0.11 µScm;^−1^ Total dissolved solid = 244 ± 0.70 mgl;^−1^ Dissolved oxygen = 5.0 ± 0.33 mg l^−1^).

Fertilization rate in this study were determined using the method and equations described by Okomoda *et al*.^32^, as shown below:

$$ \% \,{\rm{Fertilization}}=\frac{Fertilized\,eggs\,in\,the\,petri\,dish}{Total\,number\,of\,eggs\,in\,the\,petri\,dish}\times 100$$

Hatchability percentage in this study was also gotten as described below.

$$ \% \,{\rm{Hatchability}}=\frac{no.\,of\,hatched\,larvae}{total\,no.\,of\,spawned\,eggs}\times 100$$

Upon determination of the hatching rates, the larvae from the control and treatment groups were reared according to the different treatments in separate aquarium tanks of 0.5 × 0.5 × 0.5 m^3^, under similar laboratory conditions for four weeks in triplicates. During this time, the larvae were first fed freshly hatched *Artemia nauplii ad libitum* post endogenous feeding for two weeks, followed by a commercial starter diet of 45% crude protein until larvae became a month old. The survival and triploidy percentages were then determined. To obtain the erythrocyte, blood was collected from the caudal peduncle of ten fish using an 18 gauge needle fitted with a heparinized syringe. A dry blood smear was then prepared on a slide according to the method previously specified by Normala *et al*.^14^, and Felip *et al*.^26^. The erythrocytes in the slide were observed under a Nikon Eclipse 80i compound microscope at 100× magnification. Following Normala *et al*.^14^, erythrocyte on the slide was section into five different blocks, ten of which were measured for each one block (n = 50 for each slide) making a total of five hundred erythrocytes measured for each group characterized. The triploidy status was then determined using the exclusive triploid range of the erythrocyte major axis previously reported for the same species which is between 11.9–14.9 μm^14^. Erythrocyte measurement below this range was considered as diploid progenies while those above were included in the triploid progenies count. Confirmation of triploidy status was also done using chromosome count following methods optimized by Okomoda *et al*.^33^ for the same species. In brief, the fish samples were injected with freshly prepared 0.05% colchicine (at the rate of 1 mlkg^−1^) solution and allowed to swim in separately aquariums for about 3 hours. Gills of the fish were removed and treated with 0.075KCL (1 hr), methanol-acetic acid fixative (three wash of 20 min each) and stained with Giemsa stain (10% for 1 hr). Prepared slides were thereafter microphotography using a Nikon Eclipse 80i compound microscope at a magnification of 100×. Chromosome identification and counting were done electronically using the Karyotyping Video Test Software (Version 3.1). Chromosome number of triploid were 3n = 84 while diploid progenies below were 2n = 56.

Data from the three breeding trials were pool together (n = 3; i.e. each trial was used as replicates) and analyzed using MINITAB 14 computer software. Firstly, descriptive statistics were done for the breeding parameters such as fertilization, hatchability, survival and triploidy percentages. Means were then separated using Fisher’s least significant differences. The Student T-test was however used to separate mean erythrocyte major axis size of diploid and triploid progenies.

## Results and Discussion

The fertilization percentages of the various treatments, as well as the control batches of eggs, were similar (between 94 to 98%) in this study (Table 1). This was somehow expected because the eggs and sperm used for breeding were from the same set of broodstocks. According to Ola-Oladimeji^34^, the similarities in fertilization of treatment egg are clear indications of similarities in the quality of gametes used. Also, following the thoughts of Hassan *et al*.^19^, the consequential effect of the shock process used for triploidization could only be evident after the treatment application. Hence, since fertilization was done three minutes before the electric shock, the negative effects of the shock process could not have been expressed before the shock was applied. The 5 ppt salinity medium used in the treatment group also seems not to have affected the outcome of the current study as there were no significant differences in all the parameters measured for the negative and the positive control (Table 1). This is in line with the findings of Hassan *et al*.^19^, and Rodriguez-Montes *et al*.^35^ who reported that low (5 ppt) and higher salinity concentration (up to 65 ppt) did not affect breeding performance of incubated red hybrid tilapia eggs. However, these findings do not invalidate the theory that salinity could be a potential shock process for the induction of triploidization in eggs of cultured fishes.

**Table 1:** Breeding characteristics and triploidization percentage of *C. gariepinus* eggs exposed to the different protocols of electric shock.

Treatment	Shock duration (min)	Fertilization rate (%)	Hatching rate (%)	Survival rate (%)	Triploidy rate (%)
9 V	3	96.10 ± 0.73	60.80 ± 3.77^b^	96.70 ± 0.16^a^	30.90 ± 0.17^d^
9 V	5	94.20 ± 0.21	60.90 ± 2.08^b^	98.40 ± 0.16^a^	30.43 ± 1.12^d^
9 V	10	94.10 ± 0.24	41.40 ± 1.44^e^	97.60 ± 0.07^a^	20.63 ± 0.95^e^
12 V	3	98.40 ± 0.11	58.20 ± 2.08^c^	97.30 ± 0.30^a^	10.43 ± 1.07 ^f^
12 V	5	95.20 ± 0.24	53.60 ± 1.24^d^	96.40 ± 1.07^a^	20.63 ± 0.90e
12 V	10	96.34 ± 0.14	56.40 ± 0.09 ^cd^	95.10 ± 0.31^a^	50.63 ± 0.25^c^
21 V	3	95.20 ± 0.40	27.20 ± 0.12 ^f^	95.40 ± 2.11^a^	70.63 ± 0.15^b^
21 V	5	96.15 ± 0.13	21.10 ± 2.08 ^g^	94.10 ± 1.76^a^	85.43 ± 0.17^a^
21 V	10	97.30 ± 0.44	1.70 ± 1.44 ^h^	70.00 ± 1.07^b^	*
+ ve Control	^—^	95.20 ± 0.02	65.50 ± 0.00^a^	96.9 ± 0.11^a^	00.00 ± 0.00 ^g^
− ve Control	^—^	95.51 ± 0.04	63.90 ± 0.00^a^	94.20 ± 0.08^a^	00.00 ± 0.00 ^g^

Numbers are means ± standard errors. *Unable to assess triploidy percentage due to poor hatching and insufficient sample size (n = 2) after two weeks of culture post-hatching. Mean in the same row with different superscripts differ significantly (Anova, P ≤ 0.05).

The induction of triploidy through electric shock was affirmed in the current study as all tested treatments produced progenies with triploidy status at varying percentages. Triploidy ranges for all treatment were between 10–85%. A much earlier study by Cadoret^23^ had reported triploidy rates of between 3–55% when oysters and mussels were exposed to an electric field strength of 600 V cm^−1^ at different duration. The findings of Teskeredvić *et al*.^25^, also showed that a co-administered electric current of 10 V and a temperature of 26 °C as a shock in coho salmon *O. kisutch* resulted into 100% triploidy induction better than when the shocks were applied alone. Recently, Hassan *et al*.^19^, reported triploidization success with electroporation to range between 29 and 93% in red hybrid tilapia (*O. mossambicus × O. niloticus*) when 12 V were applied at varying durations. The suppression of the extrusion of the second polar body has been explained in different researches using different theories. Researchers like Piferrer *et al*.^4^, Pandian and Koteeswaran^12^, and Maxime^15^, had respectively suggested alterations in developmental rates; disruption of the microtubules of the meiotic spindle and induced cytoplasmic density changes as the underlying mechanisms of triploidy induction. While the underlining mechanism for electric shock remains unclear, the effect of the shock could have been expressed in any of the mechanisms described above.

The findings of this study showed a consistent trend of decrease in hatchability and an increase in triploidy rate with increased electroporation voltages/shock durations (Table 1). Hence, higher electric shock and durations were more efficient for triploid induction but resulted in lower rates of survival. Peruzzi *et al*.^36^, Galbreath and Samples^20^ had shown that reduction in thermal shock intensity and duration favored hatchability but decreased the number of triploids obtained in the Atlantic cod *Gadus morhua* and Brook Trout *Salvelinus fontinalis* respectively. Similarly, longer durations of hydrostatic pressure shocks caused high triploidy induction and mortality in Nile tilapia, *O. niloticus*^37^. This same phenomenon has played out in the production of triploid salmonids using thermal shock in many previous studies [e.g.^38–40^]. The various observations in all these studies suggest that beyond an optimal point, an increase in shock intensity or its duration becomes detrimental to the survival of the eggs. Oliver *et al*.^41^, had observed that beyond the optimal thermal shock of 16 °C at 120 °C minutes post-fertilization for 500 °C minutes, survival and hatchability reduced while higher percentages of triploid were observed in Burbot *Lota lota*. This is in contrast with the findings of Teskeredvić, *et al*.^25^, who reported exceptionally high survival (63–98%) and triploidy percentage (100%) with prolonged intervals of co-administered electric and temperature shock in coho salmon *O. kisutch*. Although shock from 21 V for 5 min was most effective in inducing triploidization (85%), hatchability, in contrast, was about the lowest in this study (21%). Pradeep *et al*.^42^, had earlier opined that pursuing the treatment with maximal triploid rates irrespective of the survival of the embryo will not be an economical strategy for aquaculture practices. Thus, it may be best to select the suitable shock treatment that can give a reasonably high triploidy rate and a substantially better hatchability of embryos^38^. Therefore, the application of 12 V for 10 mins in this study seems to be better for the induction of triploidy in *C. gariepinus* as it gave 50% triploidy and 56.4% hatchability.

The inability to achieve a 100% triploidy rate in this study even at higher treatments may be suggestive that the electric shock protocol had not been optimized or the shock process isn’t effective as other shock protocols previously reported. Teskeredvić *et al*.^24^, had earlier stated that uneven distribution of trauma is the resultant cause of low triploidy induction success besides other factors like egg size and quality. The above assumption could be true for the current study. This is because the electric field applied in the upper layer of the incubation chamber may not have efficiently reached all the eggs due to the plastic strainers used as hatching substrates in the study. Future studies can then design iron strainers connected directly to the battery terminals for better electric transmission to the eggs. Many previous studies have also suggested that the optimum time of shock initiation post-fertilization may not be uniform for different shock protocols^36,43^. In the study by Linhart *et al*.^43^, the second polar body was withheld at six minutes after fertilization using four minutes hydrostatic pressure shock treatments of 600 kg cm^–2^ and resulting in 100% triploidy induction of European catfish. However, using a heat-shock of 41 °C for one minute and at nine minutes after fertilization resulted in 100% triploidy in the same species^43^. These finding, therefore, shows that the time required for initiation of shock treatment was lower using pressure shock compared to heat-shock. Similarly, in *O. niloticus*, Hussain *et al*.^37^ found out that to achieve 100% triploidy induction; pressure shock needed a longer post-fertilization initiation time of nine minutes; cold-shock, however, was seven minutes while the lowest time was observed for heat-shock at five minute post-fertilization. Hence, the non-optimization of the timing of shock initiation in the current study may add to why a 100% triploidy percentage was not attained in the treated fish. To this effect, the current study may not have presented a complete account of the best protocol for electroporation in African catfish egg. However, since indiscriminate reproduction is not a problem in Catfish culture, the findings of this study are of great importance as a large proportion of the progenies is likely to display growth advantage following the observation of triploids performance in many previous studies. More studies will be needed to achieve 100% triploidy using electroporation.

**Figure 1:**
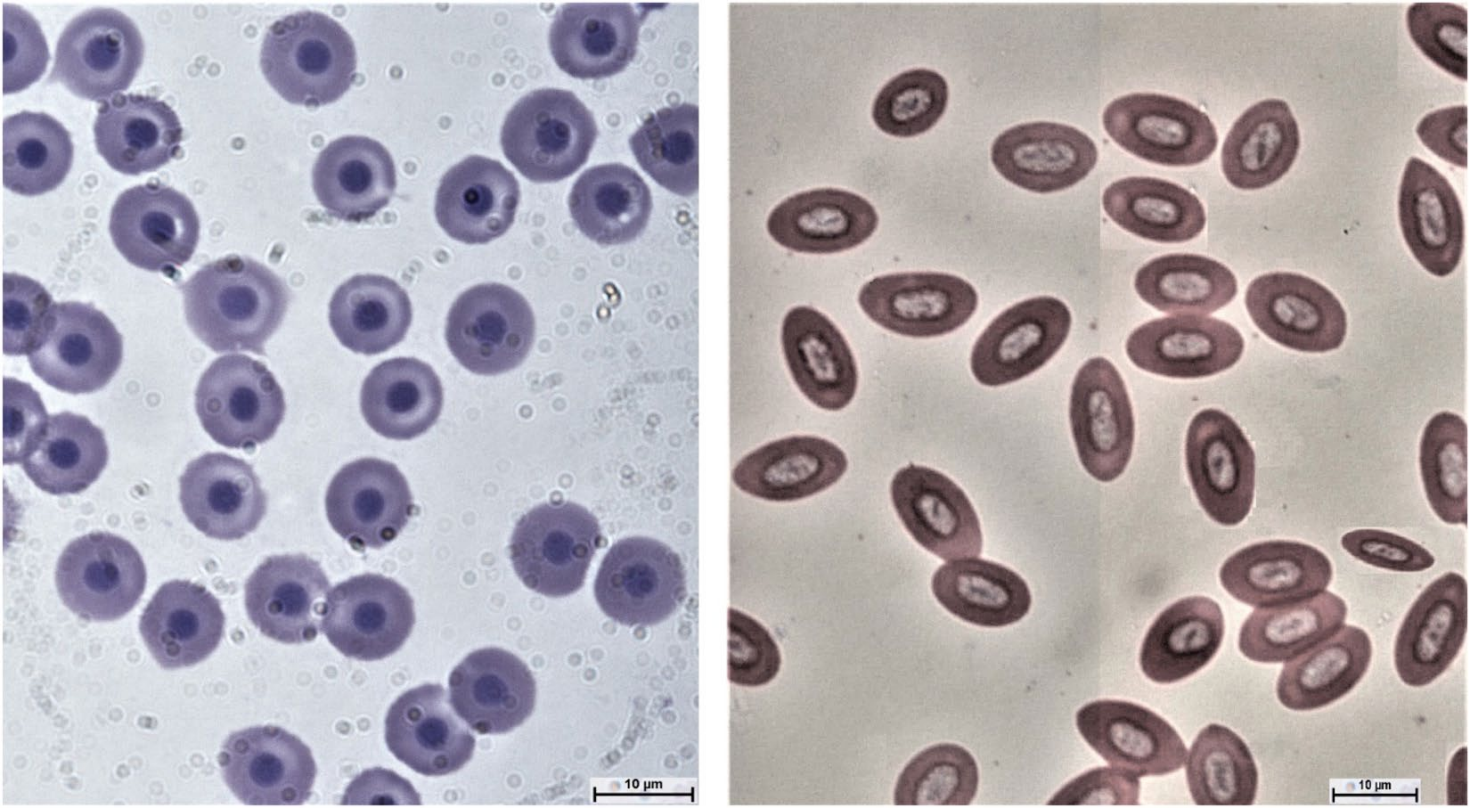
Erythrocyte morphology of diploid (left) and triploid (right) *C. gariepinus*. Bar = 10 µm.

**Figure 2:**
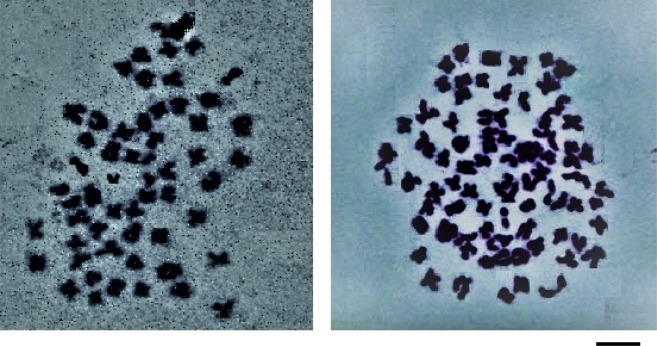
Chromosome of diploid (left) and triploid (right) *C. gariepinus* (2n = 56 and 3n = 84 respectively). Bar = 5 µm.

Erythrocyte characteristic has earlier been proven to be the simplest index of discrimination between triploid and diploid progenies. Although the erythrocytes of the triploid were visibly oval shaped compared to the diploids which were rounded (Figure 1), the use of the exclusive triploid range of erythrocyte major axis was perceived to be better and very accurate indices of discrimination in this study. This is because 100% of the erythrocytes sampled were all within the range (11.3–14.6 μm) and conformed to the expectation of triploids been 1.5 times (mean length of 13.12 ± 0.74 μm) the size of diploid counterparts (range between 7.0–10.5 μm; mean length of 9.71 ± 1.03 μm) as previously observed in many studies^13,29,44^. It is also important to state that these fish had a chromosome count of 3n = 84 (Figure 2). Although, some studies had identified polyploidy in fishes solely using the erythrocyte major axis^18,45,46^, only recently was the establishment of “exclusive triploid range” proposed by Normala *et al*.^14^. It has since been established in triploid red hybrid tilapia and found easier to discriminate triploid from diploids^18^. From the result of this study, it is concluded that electroporation can be used in triploidy induction of African catfish, *C. gariepinus*.
